# Genome Sequences of Three hpAfrica2 Strains of *Helicobacter pylori*

**DOI:** 10.1128/genomeA.00729-13

**Published:** 2013-09-26

**Authors:** Stacy S. Duncan, M. Teresita Bertoli, Dangeruta Kersulyte, Pieter L. Valk, Sravya Tamma, Issy Segal, Mark S. McClain, Timothy L. Cover, Douglas E. Berg

**Affiliations:** Department of Medicine, Vanderbilt University School of Medicine, Nashville, Tennessee, USAa; Department of Molecular Microbiology, Washington University Medical School, St. Louis, Missouri, USAb; Department of Gastroenterology, Chris Hani Baragwanath Hospital, Johannesburg, South Africac; Department of Pathology, Microbiology and Immunology, Vanderbilt University School of Medicine, Nashville, Tennessee, USAd; Veterans Affairs Tennessee Valley Healthcare System, Nashville, Tennessee, USAe; Division of Infectious Disease, Department of Medicine, University of California, San Diego, La Jolla, California, USAf

## Abstract

We present the genome sequences of three hpAfrica2 strains of *Helicobacter pylori*, which are postulated to have evolved in isolation for many millennia in people of San ethnicity. Although previously considered to be ancestral to *Helicobacter acinonychis*, the hpAfrica2 strains differ markedly from *H. acinonychis* in their gene arrangement. These data provide new insights into *Helicobacter* evolution.

## GENOME ANNOUNCEMENT

*Helicobacter pylori* chronically colonizes the stomachs of billions of people worldwide, sometimes leading to gastroduodenal disease, although usually without symptoms and sometimes with beneficial effects (1). *H. pylori* is very diverse genetically, with different sets of genotypes predominating in well-separated human populations (2, 3). Two superlineages have been described: one including hpNEAfrica, hpAfrica1, and all non-African *H. pylori* strains and one including hpAfrica2, which is postulated to have diverged from mainstream *H. pylori* some 100,000 years ago and evolved in isolation in southern African San (hunter-gatherer, click-speaking) societies (4). We sequenced the genomes of three strains classified by multilocus sequence typing (MLST) as being of the unusual hpAfrica2 type, found among 17 strains cultured with informed consent from patients from Soweto in Chris Hani Baragwanath Hospital, Johannesburg, South Africa, from 1997 to 1999. Notably, these patients were of Bantu, not San, ethnicity.

DNAs from single colonies were sequenced using 454 methods (read lengths, ≥400 bp; coverage, ≥25-fold) and assembled with 454 Newbler software. All gaps between contigs were filled by PCR and capillary sequencing (5), and sequences were annotated at NCBI (http://www.ncbi.nlm.nih.gov/genome/annotation_prok/; strain SouthAfrica7) or with the Institute for Genome Sciences (IGS) Annotation Engine (http://ae.igs.umaryland.edu/cgi/index.cgi) (6). The three SAfrica strain genome sizes are 1.6 to 1.7 Mb, which is typical for *H. pylori*.

A BLASTn analysis of sequential 1-kb fragments genome-wide indicated that these SAfrica strains are closely related to one another, with little if any admixture from non-hpAfrica2 strains. nWayComp analysis (7) identified 173 open reading frames (ORFs) in *Helicobacter acinonychis* (GenBank accession no. NC_008229.1, postulated to descend from hpAfrica2) (4) that are absent in the SAfrica strains and, conversely, 93 ORFs in the SAfrica strains that are absent from *H. acinonychis*. One gene present in all three SAfrica strains (HPSA_05790, encoding the DnaJ-like heat shock protein) had no close homolog in the current NCBI database, and another (HPSA_06345, hypothetical) was absent from all but one other *H. pylori* strain. Each SAfrica strain lacks the *cag* pathogenicity island and contains a type II *hopQ* outer membrane protein gene. The SAfrica strains contain s2/m2-type *vacA* genes (although *vacA* in SouthAfrica7 is frameshifted). These features are associated with low gastroduodenal disease risk.

Mauve analysis (8) illustrated that the *H. pylori* genome organization is well conserved, with <10 rearrangements (inversions/translocations, defined as locally colinear blocks; http://gel.ahabs.wisc.edu/mauve/) among the SAfrica strains and <20 such differences between the SAfrica and non-hpAfrica2 strains, such as J99. However, >100 rearrangements distinguished *H. acinonychis* from these putatively ancestral hpAfrica2-type strains. The SAfrica strains lack all but one *H. acinonychis* prophage gene (a gene also present in many prophage-free *H. pylori* strains and thus probably not of viral origin).

Collectively, these outcomes heighten interest in the proposed ancestry relationships between *H. pylori* and *H. acinonychis* (4) and more generally between the many helicobacters of humans and other mammals. Further analyses of hpAfrica2 genomes should provide new insights into important evolutionary questions—for example, how bacterial superlineages and distinct species (here, hpAfrica2 from ethnic Bantu Soweto residents, and *H. acinonychis*) arose and diverged from ancestral forms and remained distinct genome-wide—especially for species such as *H. pylori*, generally considered to enjoy “free recombination” (9).

### Nucleotide sequence accession numbers.

The genome sequences of these *H. pylori* strains have been deposited in GenBank under the following accession no.: SouthAfrica7, CP002336; SouthAfrica20, CP006691; and SouthAfrica50, AVNI00000000. The versions described in this paper are the first versions (SouthAfrica7, CP002336.1; SouthAfrica 20, CP006691.1; and SouthAfrica 50, AVNI01000001.1 and AVNI01000002.1).
